# Humoral and Cellular Vaccination Responses against SARS-CoV-2 in Hematopoietic Stem Cell Transplant Recipients

**DOI:** 10.3390/vaccines9101075

**Published:** 2021-09-25

**Authors:** Monika Lindemann, Vesna Klisanin, Laura Thümmler, Neslinur Fisenkci, Nikolaos Tsachakis-Mück, Markus Ditschkowski, Sina Schwarzkopf, Hannes Klump, Hans Christian Reinhardt, Peter A. Horn, Michael Koldehoff

**Affiliations:** 1 Institute for Transfusion Medicine, University Hospital Essen, University Duisburg-Essen, 45147 Essen, Germany; laura.thuemmler@stud.uni-due.de (L.T.); neslinurfisenkci@gmail.com (N.F.); sina.schwarzkopf@gmx.de (S.S.); hannes.klump@uk-essen.de (H.K.); peter.horn@uk-essen.de (P.A.H.); 2 Department of Hematology and Stem Cell Transplantation, University Hospital Essen, University Duisburg-Essen, 45147 Essen, Germany; vesna.klisanin@uk-essen.de (V.K.); nikolaos.tsachakis-mueck@uk-essen.de (N.T.-M.); markus.ditschkowski@uk-essen.de (M.D.); christian.reinhardt@uk-essen.de (H.C.R.); michael.koldehoff@uk-essen.de (M.K.)

**Keywords:** SARS-CoV-2, vaccination, allogeneic hematopoietic stem cell transplantation, antibodies, ELISpot, sex-dependency

## Abstract

The cellular response to SARS-CoV-2 vaccination and infection in allogeneic hematopoietic stem cell transplant (HSCT) recipients is not yet clear. In the current study, HSCT recipients prior to and post vaccination were tested for SARS-CoV-2-specific humoral and cellular immunity. Antibodies against spike (S) 1 were assessed by Anti-SARS-CoV-2 IgG ELISA (Euroimmun). Cellular immunity was analyzed by an *in house* interferon-gamma ELISpot and T-SPOT.*COVID* (Oxford Immunotec), using altogether seven SARS-CoV-2-specific antigens. In 117 HSCT patients vaccinated twice, SARS-CoV-2 IgG antibodies were significantly higher than in HSCT controls pre vaccination (*p* < 0.0001). After the second vaccination, we observed a median antibody ratio of 4.7 and 68% positive results, whereas 35 healthy controls reached a median ratio of 9.0 and 100% positivity. ELISpot responses in patients were significantly (*p* < 0.001) reduced to ≤33% of the controls. After the second vaccination, female HSCT patients and female healthy controls showed significantly higher antibody responses than males (6.0 vs. 2.1 and 9.2 vs. 8.2, respectively; *p* < 0.05). Cellular immunity was diminished in patients irrespective of sex. In conclusion, especially male HSCT recipients showed impaired antibody responses after SARS-CoV-2 vaccination. Changing the vaccine schedule or composition could help increase vaccine responses.

## 1. Introduction

The severe acute respiratory syndrome coronavirus type 2 (SARS-CoV-2) has caused a pandemic worldwide, and the clinical course of the disease shows substantial variation. Recent reports suggested that patients with hematological disorders such as leukemia, lymphoma and autologous or allogeneic hematopoietic stem cell transplantation (HSCT) had a higher risk of contracting SARS-CoV-2 than the general population, and infected people have a higher mortality rate [1]. Although vaccinations against SARS-CoV-2 may prevent infection, its effectiveness needs to be proven in this immunocompromised cohort. In patients vaccinated after hematopoietic stem cell transplantation, specific antibody responses were impaired [2,3,4]. However, there are no reports on T cell immunity so far.

In the present study, we report on a large, twice vaccinated HSCT cohort (*n* = 117), in which T cell immunity against SARS-CoV-2 was analyzed by interferon (IFN)-γ ELISpot, using seven SARS-CoV-2-specific antigens. Moreover, the strength of immunity was compared with various HSCT controls (patients prior to vaccination, after the first vaccination and after SARS-CoV-2 infection) and with healthy controls (after the second vaccination and after infection). Finally, we analyzed if covariates, such as sex, age or interval between HSCT, vaccination or infection and testing, had an impact on SARS-CoV-2-specific immunity.

## 2. Materials and Methods

### 2.1. Volunteers

Our study includes 117 patients after allogeneic hematopoietic stem cell transplantation (HSCT), who had been vaccinated with two doses against SARS-CoV-2 (Table 1). The group contained 61 females and 56 males, and their median age was 59 years (range 21–77). Ninety-five percent of patients were vaccinated with Comirnaty^®^ (BNT162b2, Biontech/Pfizer). Immune responses in the vaccinated volunteers were analyzed at a median of 31 days (range 11–137) after the second dose. The median interval between transplantation and 2nd vaccination was 30 months (range 5–391 months).

For comparison, we tested HSCT patients prior to vaccination (*n* = 19), after the first dose (*n* = 28), after SARS-CoV-2 infection (*n* = 8) and after SARS-CoV-2 infection plus vaccination (*n* = 1). The median interval between infection and testing was 134 days (61–218, *n* = 8) or the time since infection was 230 days. Twenty-four HSCT patients were tested sequentially. Our study included a total of 153 HSCT patients (186 samples). Overall, the vaccine was well tolerated, and few side effects after vaccination were reported. The majority of HSCT patients received allogeneic peripheral blood stem cells from unrelated donors. Fifty-seven patients received immunosuppressive drugs at the time of vaccination, and 60 patients did not.

As a control group, we included 35 healthy volunteers who had been vaccinated with two doses against SARS-CoV-2 (22 female, 13 male), without symptoms of SARS-CoV-2 infection before vaccination. Their median age was 53 years (range 24–83). Immunity of the vaccinated volunteers was analyzed at a median of 30 days (range 21–77) after the second dose of the vaccination. Seventeen of them were vaccinated with the Biontech/Pfizer vaccine, sixteen with Spikevax^®^ (mRNA-1273, Moderna Biotech) and two with the vector-based vaccine Vaxzevria^®^ (AstraZeneca) followed by the Moderna vaccine. A further control group comprised volunteers without transplantation or immunosuppression, either with acute SARS-CoV-2 infection (*n* = 17) or with resolved SARS-CoV-2 infection (and willing to donate convalescent plasma (*n* = 27)). The median interval between acute or resolved infection and testing was 13 days (2–22) or 77 days (24–394), respectively.

The study was conducted according to the guidelines of the Declaration of Helsinki, and approved by the Ethics Committee of the University Hospital Essen, Germany (20-9225-BO, 20-9254-BO and 20-9256-BO). Informed consent was obtained from all subjects involved in the study.

### 2.2. Antibody ELISA

Antibodies were determined by a CE marked Anti-SARS-CoV-2 IgG semi-quantitative ELISA (Euroimmun, Lübeck, Germany), according to the manufacturer’s instructions. Results of the S1 protein-specific IgG antibodies are given as ratio (patient sample/control sample). An antibody ratio of ≥1.1 was considered positive, of ≥0.8 to <1.1 borderline and of <0.8 negative.

### 2.3. ELISpot Assay

To assess SARS-CoV-2-specific cellular immunity, we performed ELISpot assays, using peptide pools of the spike (S) 1, the S1/S2, the membrane (M) and the nucleocapsid (NC) proteins (Miltenyi Biotec, Bergisch Gladbach, Germany) and an S1 protein of SARS-CoV-2 (S Sino, Val 16–Arg 685, Sino Biological, Wayne, PA, USA). In 61 samples, we performed a CE-marked, commercial SARS-CoV-2 ELISpot, the T-SPOT.*COVID* (Oxford Immunotec, Abingdon, Oxfordshire, United Kingdom) in parallel. This commercial ELISpot includes a peptide mix of the SARS-CoV-2 spike and NC. We tested 250,000 peripheral blood mononuclear cells (PBMC) per sample and measured IFN-γ production after 19 h of incubation. SARS-CoV-2-specific spots were determined as stimulated minus non-stimulated (background) values (spots increment). In an ELISpot established *in house*, we defined at least three spots above background together with threefold higher SARS-CoV-2-specific spots versus background as positive response. Thereby, the detection limit of this assay is in the range of 3/250,000 SARS-CoV-2-specific cells, which secrete IFN-γ. Details on this ELISpot assay and the cutoff definition have been published previously [5]. For the T-SPOT.*COVID*, we used the cutoff given by the manufacturer (6 spots increment).

### 2.4. Statistical Analysis

Statistical analysis was performed with GraphPad Prism 8.0.1 (San Diego, CA, USA) and IBM SPSS Statistics 23 (New York, NY, USA) software. For the analysis of numerical variables, we used Spearman correlation and linear regression analysis. To assess the impact of categorical covariates, we used Mann–Whitney or Kruskal–Wallis test with Dunn’s multiple comparisons test, as appropriate. Two-sided *p* values < 0.05 were considered significant.

## 3. Results

### 3.1. Comparison of Vaccination Responses in Patients after Hematopoietic Stem Cell Transplantation and Healthy Controls

**Figure 1:**
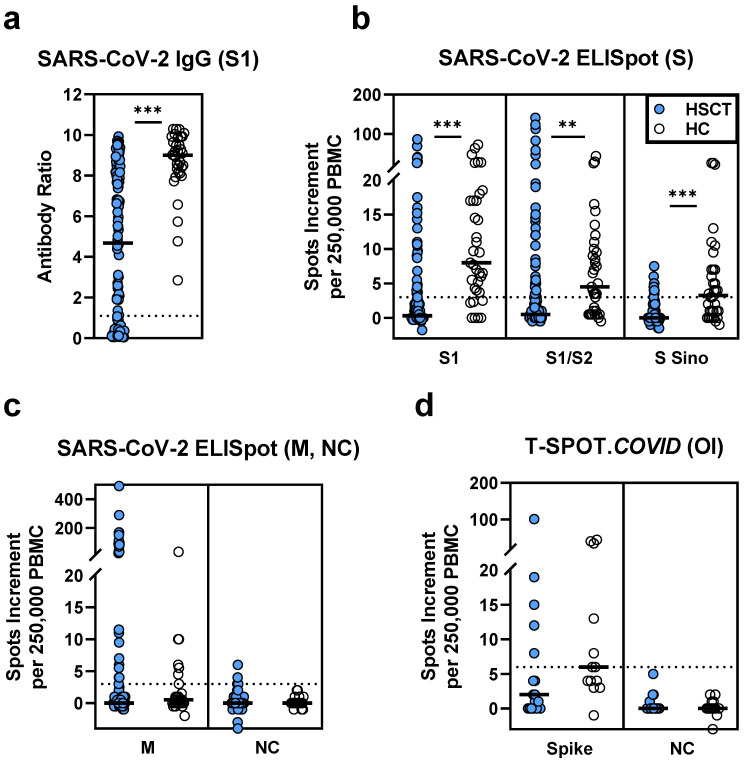
SARS-CoV-2-specific IgG and ELISpot responses in 117 patients after hematopoietic stem cell transplantation (HSCT) and 35 healthy controls (HC), after two SARS-CoV-2 vaccinations. Panel (**a**) shows IgG antibody responses against SARS-CoV-2 spike (S) 1, (**b**) ELISpot responses to S antigens (S) and (**c**) to membrane (M) and nucleocapsid (NC). For comparison to these *in house* ELISpot assays (**b**,**c**), results of a commercial ELISpot, the T-SPOT.*COVID* (Oxford Immunotec, OI), are shown as panel (**d**). Parallel tests with both ELISpot formats were, performed in 17 patients and 13 healthy controls, respectively. Horizontal bold lines indicate median values, and dashed lines the cutoff for positive responses (antibody ratio of 1.1, 3 spots increment for *in house* ELISpot assays and 6 spots increment for the T-SPOT.*COVID*). S1-peptide mix of the SARS-CoV-2 spike (S) 1; S1/S2-peptide mix of the spike (S) 1 and S2; S Sino-S1 protein (Sino Biological); M-peptide mix of the membrane; NC-peptide mix of the nucleocapsid. ** *p* = 0.0002, *** *p* < 0.0001 (Mann–Whitney test).

In 117 HSCT patients vaccinated twice against SARS-CoV-2, IgG antibody levels directed against the S1 antigen were significantly lower than in 35 healthy controls (median antibody ratio of 4.7 vs. 9.0, *p* < 0.0001, Figure 1a). Their T cell responses were also significantly diminished (*p* ≤ 0.0002). In particular, responses to various SARS-CoV-2 spike antigens were reduced to a third or less (≤33%) of healthy controls (S1: 0.5 vs. 8.0; S1/S2: 0.5 vs. 4.5; S Sino: 0 vs. 3.5, spike (T-SPOT.*COVID*): 2.0 vs. 6.0; data represent median values of spots increment, Figure 1b,d). Of note, the observed differences between HSCT patients and healthy controls cannot be attributed to the different SARS-CoV-2 vaccines used. Considering only healthy controls who received the Biontech/Pfizer vaccine (*n* = 17), we observed a median antibody ratio of 8.3 and similar numbers of spots increment as in all 35 healthy volunteers (S1: 8.0; S1/S2: 3.5; S Sino: 3.0; spike (T-SPOT.*COVID*): 6.0).

In the control experiments using nucleocapsid (NC) peptides, three HSCT patients showed detectable ELISpot responses, whereas none of the healthy controls responded (Figure 1c). Thus, three patients may have been infected with SARS-CoV-2, although they had no clinical evidence of a previous SARS-CoV-2 infection. Responses to a rather conserved coronavirus antigen, the membrane protein (M), were also similar in both cohorts.

**Figure 2:**
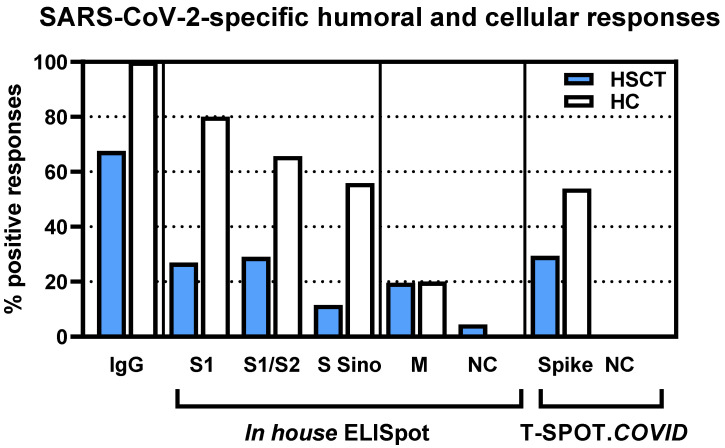
Percentage of positive responses in 117 patients after hematopoietic stem cell transplantation (HSCT) and in 35 healthy controls, after two SARS-CoV-2 vaccinations. We used the following cutoff values for positivity: antibody ratio of 1.1, 3 spots increment for *in house* ELISpot assays and 6 spots increment for the T-SPOT.*COVID* (Oxford Immunotec). The T-SPOT.*COVID* was performed only in a subset of volunteers (17 HSCT patients, 13 healthy controls). IgG-IgG directed against the S1 protein of SARS-CoV-2; S1-peptide mix of the SARS-CoV-2 spike (S) 1; S1/S2-peptide mix of the spike (S) 1 and S2; S Sino-S1 protein; M-peptide mix of the membrane; NC-peptide mix of the nucleocapsid.

Calculating the number of positive SARS-CoV-2-specific humoral or cellular responses, the percentage was also reduced in the HSCT patients (Figure 2). Detectable SARS-CoV-2 antibodies were observed in 68% of the patients vs. 100% of the controls. The percentage of patients with detectable T cell immunity was reduced to 21–55% of the controls, depending on the spike antigen employed for stimulation (S1: 27 vs. 80%; S1/S2: 29 vs. 66%; S Sino: 12 vs. 56%; spike (T-SPOT.*COVID*): 29 vs. 54%).

### 3.2. Correlation of SARS-CoV-2-Specific Immunity Measured by Various Assays

Spearman correlation analysis indicated that SARS-CoV-2-specific antibodies in patients after two vaccinations correlated significantly with their T cell activities as determined by ELISpot responses, especially after stimulation with the peptide mix S1 (*r* = 0.45, *p* < 0.0001) and with the spike peptides of T-SPOT.*COVID* (*r* = 0.52, *p* = 0.04) (Table 2). Of note, the correlation was weaker in the healthy controls. Whereas all healthy controls showed strong antibody responses, only 54–80% (depending on the antigen) had detectable SARS-CoV-2-specific T cells.

Furthermore, we performed correlation analyses for the various ELISpot assays with cells from patients who had received two vaccinations (Table 3). We observed the highest correlation coefficient for responses to the S1 peptide mix of the *in house* ELISpot and to the spike peptides of T-SPOT.*COVID* (*r* = 0.53, *p* = 0.03).

The combined analysis of a total of 265 samples from HSCT patients (prior to and post vaccination and post infection, *n* = 186), healthy controls after the second vaccination (*n* = 35) and controls after acute or resolved SARS-CoV-2 infection (*n* = 44) indicated the strongest correlation between ELISpot responses against the S1 and the S1/S2 peptide mix (*r* = 0.55, *p* < 0.0001) and between ELISpot responses against the S1 peptide mix and S Sino (*r* = 0.51, *p* < 0.0001). Responses against the S1 peptide mix of the *in house* ELISpot and the spike peptides of T-SPOT.*COVID* showed significant correlation (*r* = 0.57, *p* < 0.0001). Moreover, responses to the NC peptides of the *in house* and commercial ELISpot showed significant correlation (*r* = 0.28, *p* = 0.03). Detailed results of the Spearman analyses on SARS-CoV-2-specific humoral and cellular immunity are presented as Supplementary Table S1.

### 3.3. Comparison of SARS-CoV-2-Specific Immunity in Stem Cell Transplant Recipients Prior to and Post Vaccination and after Infection

**Figure 3:**
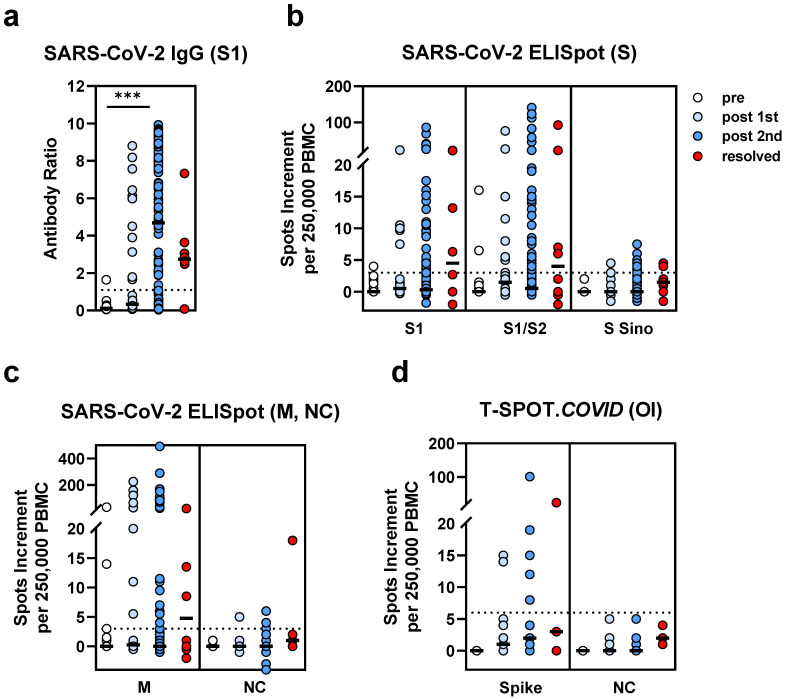
SARS-CoV-2-specific IgG and ELISpot responses in hematopoietic stem cell transplant recipients. We compared data in patients prior to SARS-CoV-2 vaccination (pre, *n* = 19), after the first vaccination (post 1st, *n* = 28), after the second vaccination (post 2nd, *n*= 117) and after infection (resolved, *n* = 8). Panel (**a**) shows IgG antibody responses against SARS-CoV-2 spike (S) 1, (**b**) ELISpot responses to S antigens (S) and (**c**) to membrane (M) and nucleocapsid (NC). For comparison with these *in house* ELISpot assays (**b**,**c**), results of the T-SPOT.*COVID* (Oxford Immunotec, OI) are shown as panel (**d**). Parallel tests with both ELISpot formats were performed in 25 patients. Horizontal bold lines indicate median values, and dashed lines the cutoff for positive responses (antibody ratio of 1.1, 3 spots increment for *in house* ELISpot assays and 6 spots increment for the T-SPOT.*COVID*). S1-peptide mix of the SARS-CoV-2 spike (S) 1; S1/S2-peptide mix of the spike (S) 1 and S2; S Sino-S1 protein (Sino Biological); M-peptide mix of the membrane; NC-peptide mix of the nucleocapsid. *** *p* < 0.0001 (Kruskal–Wallis test).

As one might expect, we observed the lowest humoral SARS-CoV-2-specific response prior to vaccination (*n* = 19), an intermediate response after the first dose of the vaccination (*n* = 28) and the strongest response after the second dose (*n* = 117) (Figure 3a). The median ratio was 0.1 prior to vaccination, 0.3 after the first and 4.7 after the second vaccination (*p* < 0.0001 for the comparison pre vs. after the second vaccination). Patients who had been infected with SARS-CoV-2 (*n =* 8) showed antibody responses which were overall between those after the first and second doses of the vaccination (median ratio of 2.7).

Cellular responses did not differ significantly between the four HSCT groups (Figure 3b–d). We observed a median spots increment to the S1 peptide mix of 0, 0.5 and 0.5 prior to vaccination, after the first dose and after the second dose, respectively. The numbers for the S1/S2 peptide mix were 0, 1.5 and 0.5 and for the spike peptides of the T-SPOT.*COVID* 0, 1 and 2, respectively. However, after infection, the spot increment was overall higher than after vaccination (S1: 4.5; S1/S2: 4; spike (T-SPOT.*COVID*): 3).

After the first dose of the vaccine, positive humoral responses against the S antigen of SARS-CoV-2 were observed in 37% of the patients and cellular responses in 7–30% of the patients (depending on the antigen used for stimulation). After infection, positive humoral responses against the S antigen of SARS-CoV-2 were observed in 75% and cellular responses in 29–50% of the patients. Interestingly, the HSCT patient tested on day 230 after SARS-CoV-2 infection and day 8 after the second vaccination showed strong humoral and cellular responses (antibody ratio of 9.8 and 86 or 25 spots increment to the S1 or NC ELISpot, respectively).

### 3.4. Comparison of SARS-CoV-2-Specific Immunity in Stem Cell Transplant Recipients and Controls after SARS-CoV-2 Infection

**Figure 4:**
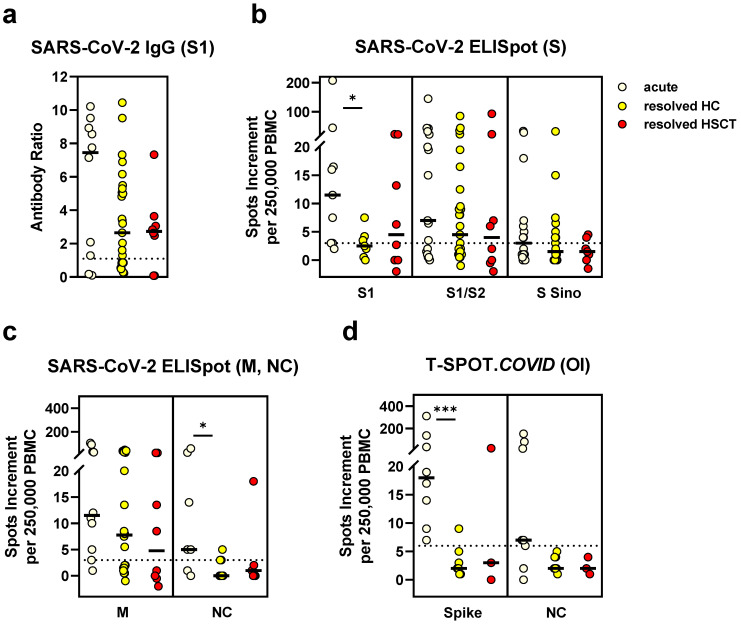
SARS-CoV-2-specific IgG and ELISpot responses in hematopoietic stem cell transplant (HSCT) recipients and non-immunosuppressed controls after SARS-CoV-2 infection. We compared the data in HSCT patients (resolved HSCT, *n* = 8) with those of controls with acute SARS-CoV-2 infection (*n* = 17) or with resolved SARS-CoV-2 infection (resolved HC, *n* = 27). Panel (**a**) shows IgG antibody responses against SARS-CoV-2 spike (S) 1, (**b**) ELISpot responses to S antigens (S) and (**c**) to membrane (M) and nucleocapsid (NC). For comparison with these *in house* ELISpot assays (**b**,**c**), results of the T-SPOT.*COVID* (Oxford Immunotec, OI) are shown as panel (**d**). Parallel tests with both ELISpot formats were performed in 19 volunteers. Horizontal bold lines indicate median values, and dashed lines the cutoff for positive responses (antibody ratio of 1.1, 3 spots increment for *in house* ELISpot assays and 6 spots increment for the T-SPOT.*COVID*). S1-peptide mix of the SARS-CoV-2 spike (S) 1; S1/S2-peptide mix of the spike (S) 1 and S2; S Sino-S1 protein (Sino Biological); M-peptide mix of the membrane; NC-peptide mix of the nucleocapsid. * *p* < 0.05, *** *p* < 0.001 (Mann–Whitney test for controls with acute vs. resolved infection).

Antibody and T cell responses in HSCT patients and non-immunosuppressed controls were similar after resolved infection (Figure 4). Of note, the controls with resolved infection were healthy and willing to donate convalescent plasma. We already reported some of the results obtained from their samples [5]. In the non-immunosuppressed cohort, we observed stronger humoral and cellular responses during acute vs. after resolved infection (*p* = 0.2 for antibodies; *p* = 0.03 for the S1 ELISpot; *p* = 0.04 for the *in house* NC ELISpot; *p* = 0.0008 for the spike peptides of T-SPOT.COVID).

### 3.5. Correlation of SARS-CoV-2 Immunity and Clinical Parameters

#### 3.5.1. Sex

**Figure 5:**
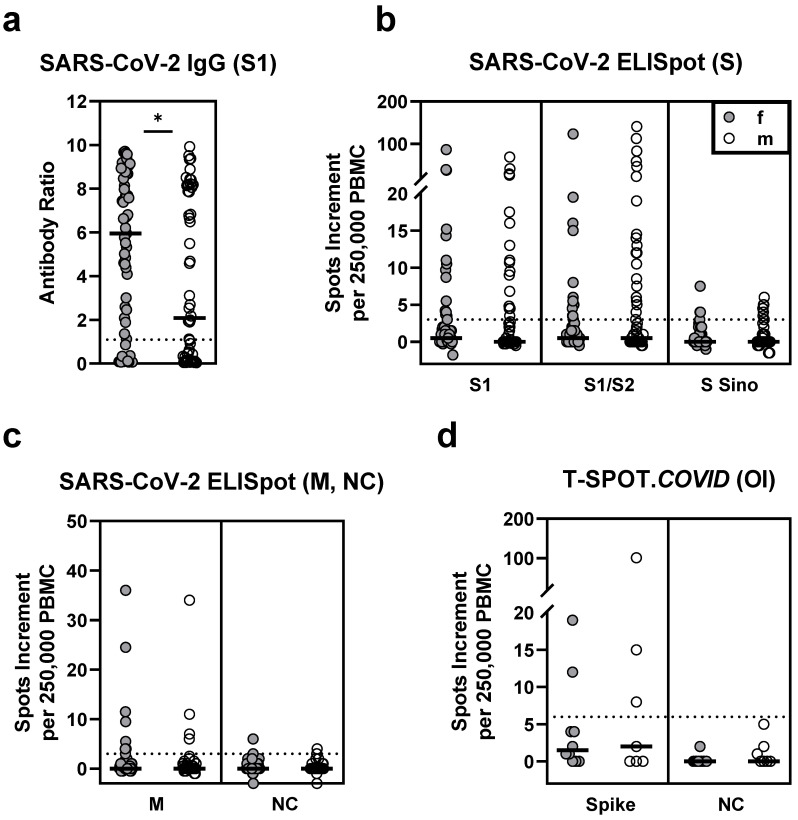
SARS-CoV-2-specific IgG and ELISpot responses in 61 female (f) and 56 male (m) patients after hematopoietic stem cell transplantation, after two SARS-CoV-2 vaccinations. Panel (**a**) shows IgG antibody responses against SARS-CoV-2 spike (S) 1, (**b**) ELISpot responses to S antigens (S) and (**c**) to membrane (M) and nucleocapsid (NC). For comparison with these *in house* ELISpot assays (**b**,**c**), results of the T-SPOT.*COVID* (Oxford Immunotec, OI) are shown as panel (**d**). Parallel tests with both ELISpot formats were performed in 17 patients. Horizontal bold lines indicate median values, and dashed lines the cutoff for positive responses (antibody ratio of 1.1, 3 spots increment for *in house* ELISpot assays and 6 spots increment for the T-SPOT.*COVID*). S1-peptide mix of the SARS-CoV-2 spike (S) 1; S1/S2-peptide mix of the spike (S) 1 and S2; S Sino-S1 protein (Sino Biological); M-peptide mix of the membrane; NC-peptide mix of the nucleocapsid. * *p* < 0.05 (Mann–Whitney test).

**Figure 6:**
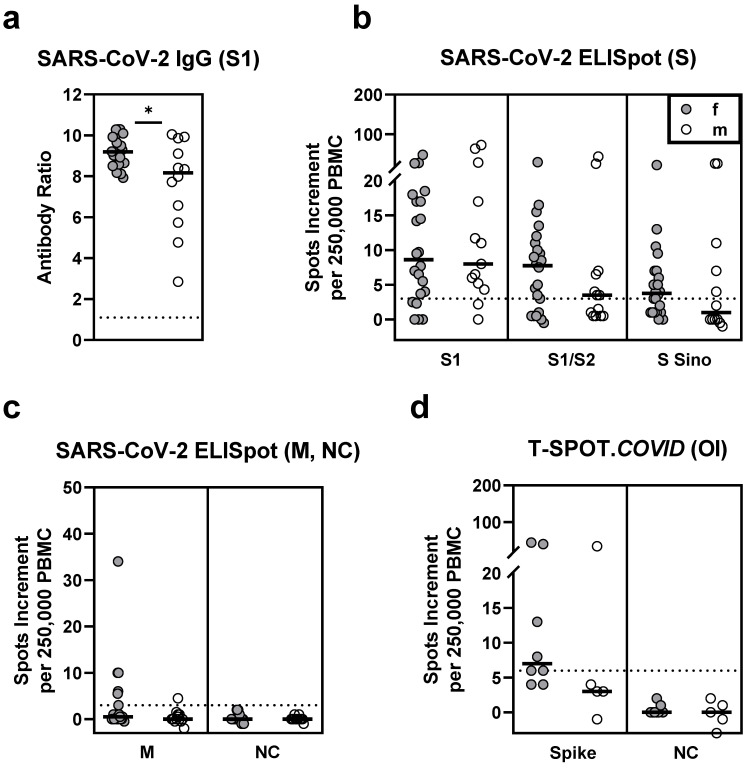
SARS-CoV-2-specific IgG and ELISpot responses in 22 female (f) and 13 male (m) healthy controls, after two SARS-CoV-2 vaccinations. Panel (**a**) shows IgG antibody responses against SARS-CoV-2 spike (S) 1, (**b**) ELISpot responses to S antigens (S) and (**c**) to membrane (M) and nucleocapsid (NC). For comparison with these *in house* ELISpot assays (**b**,**c**), results of the T-SPOT.*COVID* (Oxford Immunotec, OI) are shown as panel (**d**). Parallel tests with both ELISpot formats were performed in 13 controls. Horizontal bold lines indicate median values, and dashed lines the cutoff for positive responses (antibody ratio of 1.1, 3 spots increment for *in house* ELISpot assays and 6 spots increment for the T-SPOT.*COVID*). S1-peptide mix of the SARS-CoV-2 spike (S) 1; S1/S2-peptide mix of the spike (S) 1 and S2; S Sino-S1 protein (Sino Biological); M-peptide mix of the membrane; NC-peptide mix of the nucleocapsid. * *p* < 0.05 (Mann–Whitney test).

In HSCT patients who received two vaccinations (*n* = 117), sex had a significant impact on the antibody responses. Females had a median antibody ratio of 6.0 and males of 2.1 (*p* = 0.03) (Table 4, Figure 5). Similarly, vaccinated female healthy controls showed significantly higher antibody ratios (9.2 vs. 8.2, *p* = 0.03) (Figure 6). However, T cell responses were comparable between female and male HSCT patients and healthy controls.

#### 3.5.2. Age

After the second vaccination, patient age (range 21–77 years) negatively correlated with SARS-CoV−2 antibody levels (r = −0.22, *p* = 0.02) and showed a similar tendency in a subset of ELISpot responses (Table 5).

#### 3.5.3. Interval between Transplantation and Testing

The interval between HSCT and testing (range 6–392 months) showed positive correlation with humoral and cellular SARS-CoV-2-specific immunity (e.g., IgG ratio: r = 0.31, *p* = 0.0006; S1: r = 0.30, *p* = 0.001; spike (T-SPOT.COVID): r = 0.67, *p* = 0.004) (Table 5).

#### 3.5.4. Interval between Vaccination and Testing

In HSCT patients, the interval between the second vaccination and testing (range 11–137 days) negatively correlated with SARS-CoV-2-specific ELISpot responses (S1: r = −0.28, *p* = 0.003; S1/S2: r = −0.21, *p* = 0.02), but not with antibody levels (Table 5).

#### 3.5.5. Interval between SARS-CoV-2 Infection and Testing

In eight HSCT patients with SARS-CoV-2 infection, the time after infection (range 61–218 days) did not correlate significantly with specific humoral or cellular immunity.

**Figure 7:**
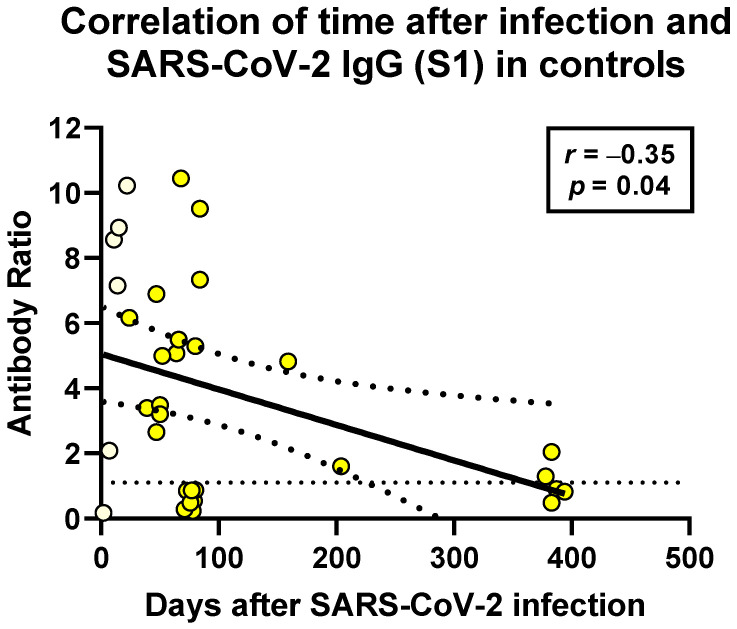
Spearman correlation analysis of SARS-CoV-2-specific IgG and distance to SARS-CoV-2 infection in non-immunosuppressed controls (*n* = 33). This analysis includes six data sets after acute infection (light yellow) and 27 after resolved SARS-CoV-2 infection (bright yellow). In the remaining controls, sera for antibody testing or information on the date of infection was not available. The horizontal dashed line indicates the cutoff for positive antibody responses (ratio of 1.1). The bold, continuous line indicates the regression line, and the two dashed lines the 95% confidence interval.

In the non-immunosuppressed controls, the time after infection (range 2–394 days) negatively correlated with humoral and cellular SARS-CoV-2-specific immunity. This finding reached statistical significance only for SARS-CoV-2-specific antibodies (r = −35, *p*=0.04) (Figure 7).

## 4. Discussion

Here, we show that both SARS-CoV-2-specific IgG antibody formation and T cell immune response are significantly impaired in hematopoietic stem cell recipients, as compared to healthy controls. In line with this finding, it is well established that vaccination responses to various pathogens, e.g., influenza virus and *Streptococcus pneumoniae*, are diminished in HSCT recipients, especially within the first 2–3 years after transplantation [6]. Specific immunity to pathogens as well as side effects to vaccination may be reduced in immunocompromised patients, e.g., after transplantation. However, this point is still controversial. One study on SARS-CoV-2 mRNA vaccination showed lower local and systemic side effects in chronically dialyzed patients, who are immunosuppressed due to impaired renal function [7]. Another group reported a number and severity of side effects in HSCT recipients similar to published data from healthy controls [8].

Whereas several reports addressed the immunogenicity of the COVID-19 vaccines in solid organ transplant recipients, especially after kidney transplantation, data after hematopoietic stem cell transplantation are still scarce. It has been published that humoral [9,10,11,12,13,14,15,16] as well as cellular vaccination responses [12,14,16] were diminished after solid organ transplantation. A recent preprint publication described that in patients after heart and lung transplantation, baseline levels of activated PD-1+ HLA-DR+ CXCR5- CD4+ T cells (also known as T peripheral helper [T _PH_] cells) and CD4+ T cells strongly predicted the ability to mount an antibody response against SARS-CoV-2 [17]. In HSCT patients without SARS-CoV-2 antibodies, low CD4+ T cell counts (<200 cells/mm^3^) were reported [18]. Nevertheless, the role of T cell immunity after vaccination in solid organ transplant recipients is controversial. A recent report by Thieme et al. [19] showed similar vaccination responses in transplant populations and non-immunosuppressed controls. In that study, T cell immunity was analyzed by intracellular cytokine staining, using flow cytometry [19]. The remaining studies on T cell immunity measured secreted IFN-γ by the ELISpot technique [12,14] or secreted IFN-γ by a whole-blood platform plus intracellular cytokines by flow cytometry [16]. A study by Herrera et al. [20] on liver and heart transplant recipients also used the ELISpot and found 79% of ELISpot positivity. Another study using intracellular cytokine staining observed specific T cells in only four out of twelve lung transplant recipients after two vaccinations [21]. However, these two later studies [20,21] did not include local healthy controls. Nevertheless, the percentage of positive ELISpot responses [20] appears as a good vaccination response, whereas a response in only 35% of vaccinated subjects [21] appears as diminished.

Results obtained from patients after hematopoietic transplantation showed that antibodies against SARS-CoV-2 were reduced after the first [2,3,4] and second vaccination [4]. Antibody responses after the second vaccination [4] were approximately 29% of healthy controls (6354 AU/mL vs. 21,395 AU/mL). In the current study, we used a different ELISA. Therefore, the results are not directly comparable. We also found a significant reduction, as compared to healthy controls (ratio of 4.7 vs. 9.0). Data on cellular SARS-CoV-2-specific responses are not yet published in the HSCT cohort. Here, we included several control groups and showed that T cell-mediated immune response is reduced to a third or less of the vaccinated healthy controls, depending on the spike antigen employed for in vitro T cell stimulation. However, T cell responses were similar in patients and in control persons after resolved infection.

Interestingly, humoral and cellular SARS-CoV-2-specific immunity tended to be stronger in HSCT patients after infection than after vaccination. This finding is in line with a recent publication, showing in a non-immunosuppressed cohort that previous infection contributes significantly to SARS-CoV-2-specific immunity and increases vaccination responses [22]. Furthermore, in a recent preprint report on vaccination responses in people over 80 years of age, a substantial increase of antibody and T cell responses after previous SARS-CoV-2 infection has been observed [23].

As already described for the general population [24], we also observed a sex-dependency of SARS-CoV-2 antibodies in HSCT patients after vaccination. Nevertheless, T cell responses did not differ significantly between females and males, neither in patients nor in healthy controls. Moreover, vaccination responses depended on age and the time point after transplantation and vaccination, as expected.

## 5. Conclusions

In male HSCT recipients, SARS-CoV-2-specific humoral vaccination responses were particularly reduced, as compared to healthy controls. In contrast, cellular responses were equally mitigated in female and male recipients. Further studies are warranted to define the best strategy to overcome this problem. Either repetitive vaccination or a modification of the vaccine could be an option, as has been demonstrated for other vaccines. For example, immunocompromised individuals are vaccinated with hepatitis B vaccines containing a higher antigen dose (40 µg HBsAg protein) to increase its immunogenicity [25], or, alternatively, highly potent adjuvants, such as monophosphoryl lipid A (MPL) and Quillaja saporina (QS21) can be used [26].

## Figures and Tables

**Table 1:** Characteristics of 117 hematopoietic stem cell transplant recipients who received two SARS-CoV-2 vaccinations.

Variable	Group	Absolute Number or Median (Range)
Sex	Female	61
	Male	56
Age (years)		59 (21–77)
Underlying disease	Acute leukemia Myelodysplastic syndromes Myeloproliferative neoplasia Lymphoma Other/not specified	70 18 14 12 3
Vaccine	Comirnaty^®^ (Biontech/Pfizer)	111
	Spikevax^®^ (Moderna Biotech)	3
	Vaxzevria^®^ (AstraZeneca)	2
	Vaxzevria^®^ followed by Comirnaty®	1
Interval transplantation- 2nd vaccination		30 months (5–391)
Interval transplantation- testing		31 months (6–392)
Interval vaccination-testing 2nd		31 days (11–137)
1st		71 days (33–158)

**Table 2:** Spearman correlation analysis of SARS-CoV-2-specific IgG antibodies and SARS-CoV-2-specific ELISpot responses in 117 patients after hematopoietic stem cell transplantation (HSCT) and in 35 healthy controls, after two SARS-CoV-2 vaccinations.

Cohort	Antigen for ELISpot	*r*	*p*
HSCT	S1	0.45	<0.0001
	S1/S2	0.22	0.02
	S Sino	0.16	0.09
	Spike (T-SPOT.*COVID*) ^1^	0.52	0.04
Healthy controls	S1	0.22	0.22
	S1/S2	0.20	0.25
	S Sino	0.40	0.02
	Spike (T-SPOT.*COVID*) ^1^	0.14	0.64

^1^ T-SPOT.*COVID* (Oxford Immunotec, OI) using the spike antigen was only performed in 17 patients and 13 healthy controls. S1-peptide mix of the SARS-CoV-2 spike (S) 1; S1/S2-peptide mix of the spike (S) 1 and S2; S Sino-S1 protein (Sino Biological).

**Table 3:** Spearman correlation of SARS-CoV-2-specific ELISpot responses in 117 patients after hematopoietic stem cell transplantation, after two SARS-CoV-2 vaccinations.

Antigen for ELISpot 1	Antigen for ELISpot 2	*r*	*p*
S1	S1/S2	0.48	<0.0001
S1	S Sino	0.34	0.0003
S1/S2	S Sino	0.11	0.24
S1	Spike (T-SPOT.*COVID*) ^1^	0.53	0.03
S1/S2	Spike (T-SPOT.*COVID*) ^1^	0.42	0.09

^1^ T-SPOT.*COVID* (Oxford Immunotec, OI) using the spike antigen was only performed in 17 out of 117 patients. S1-peptide mix of the SARS-CoV-2 spike (S) 1; S1/S2-peptide mix of the spike (S) 1 and S2; S Sino-S1 protein (Sino Biological).

**Table 4:** Influence of sex on SARS-CoV-2-specific IgG and ELISpot responses in 117 patients after hematopoietic stem cell transplantation (HSCT), after two SARS-CoV-2 vaccinations ^1^.

Test	Female (*n* = 61)	Male (*n* = 56)	*p*
IgG	6.0 (0.07–9.7)	2.1 (0.04–9.9)	0.03
S1 ELISpot	0.5 (−2–87)	0 (−0.5–69)	0.49
S1/S2 ELISpot	0.5 (−0.5–124)	0.5 (−0.5–141)	0.52
S Sino ELISpot	0 (−1–7.5)	0 (−1.5–6)	0.76
Spike (T-SPOT.*COVID*) ^2^	1.5 (0–19)	2.0 (0–101)	0.86

^1^ Data represent median (range). ^2^ T-SPOT.*COVID* (Oxford Immunotec, OI) using the spike antigen was only performed in 17 patients. S1-peptide mix of the SARS-CoV-2 spike (S) 1; S1/S2-peptide mix of the spike (S) 1 and S2; S Sino-S1 protein (Sino Biological).

**Table 5:** Spearman correlation analysis of clinical parameters and SARS-CoV-2-specific IgG and ELISpot responses in 117 patients after hematopoietic stem cell transplantation (HSCT), after two SARS-CoV-2 vaccinations.

Parameter	Test	*r*	*p*
Age	IgG	−0.22	0.02
	S1 ELISpot	−0.05	0.59
	S1/S2 ELISpot	0.06	0.55
	S Sino ELISpot	−0.10	0.28
	Spike (T-SPOT.*COVID*) ^1^	−0.31	0.23
Interval HSCT-testing	IgG	0.31	0.0006
	S1 ELISpot	0.30	0.001
	S1/S2 ELISpot	0.32	0.0004
	S Sino ELISpot	0.06	0.52
	Spike (T-SPOT.*COVID*) ^1^	0.67	0.004
Interval 2nd vaccination-testing	IgG	−0.05	0.59
	S1 ELISpot	−0.28	0.003
	S1/S2 ELISpot	−0.21	0.02
	S Sino ELISpot	−0.14	0.14
	Spike (T-SPOT.*COVID*) ^1^	−0.08	0.77

^1^ T-SPOT.*COVID* (Oxford Immunotec, OI) using the spike antigen was only performed in 17 patients. S1-peptide mix of the SARS-CoV-2 spike (S) 1; S1/S2-peptide mix of the spike (S) 1 and S2; S Sino-S1 protein (Sino Biological).

## Data Availability

The data presented in this study are available on request from the corresponding author. The data are not publicly available due to privacy restrictions.

## Supplementary Materials

The following are available online at https://www.mdpi.com/article/10.3390/vaccines9101075/s1, Table S1: Spearman correlation analysis of SARS-CoV-2-specific immunity measured by various assays.
