# Supplementary material for: Humoral and Cellular Vaccination Responses against SARS-CoV-2 in Hematopoietic Stem Cell Transplant Recipients
Source: Vaccines (Basel). 2021 Sep 25;9(10):1075. doi: 10.3390/vaccines9101075 (PMC8537291; doi:10.3390/vaccines9101075)
Supplement: Supplementary file 1 [file vaccines-09-01075-s001.zip › vaccines-1350473-supplementary.pdf]

Supplementary Table S1. Spearman correlation analysis of SARS-CoV-2-specific immunity measured by various assays.

		IgG	S1	S1/S2	S Sino	Spike (OI)	M	NC	NC (OI)
**IgG**	*r*		.43	.31	.32	.57	.04	.04	-.18
	p		< 0.0001	< 0.0001	< 0.0001	< 0.0001	0.50	.57	.19
	n		227	257	238	54	243	213	54
**S1**	*r*	.43		.55	.51	.57	.24	.32	.13
	p	< 0.0001		< 0.0001	< 0.0001	< 0.0001	< 0.0001	< 0.0001	.33
	n	227		235	226	61	235	221	61
**S1/S2**	*r*	.31	.55		.36	.43	.59	.19	.22
	p	< 0.0001	< 0.0001		< 0.0001	.0005	< 0.0001	.006	.08
	n	257	235		246	61	251	221	61
**S Sino**	*r*	.32	.51	.36		.39	.20	.17	.30
	p	< 0.0001	< 0.0001	< 0.0001		.002	0.002	.009	.02
	n	238	226	246		61	233	221	61
**Spike (OI)**	*r*	.57	.57	.43	.39		.17	.20	.36
	p	< 0.0001	< 0.0001	.0005	0.002		0.20	.13	.004
	n	54	61	61	61		61	60	61
**M**	*r*	.04	.24	.59	.20	.17		.25	.41
	p	.50	< 0.0001	< 0.0001	0.002	.20		< 0.0001	.001
	n	243	235	251	233	61		221	61
**NC**	*r*	.04	.32	.19	.17	.20	.25		.28
	p	.57	< 0.0001	.006	0.009	.13	< 0.0001		.03
	n	213	221	221	221	60	221		60
**NC (OI)**	*r*	-.18	.13	.22	.30	.36	.41	.28	
	p	.19	.33	.08	.02	.004	.001	.03	
	n	54	61	61	61	61	61	60	

This Spearman correlation analysis comprises altogether 265 samples. It contains 186 samples from 153 patients after hematopoietic stem cell transplantation (prior to and post vaccination and post infection), from 35 healthy controls after the second vaccination and from 44 controls after acute or resolved SARS-CoV-2 infection. Please note that sample numbers vary because at the beginning of our study only a subset of SARS-CoV-2-specific antigens were available and because in some volunteers serum samples were lacking. Light yellow labelling indicates $p < 0.05$ and bright yellow $p < 0.0001$. *r*-Spearman correlation coefficient; S1-peptide mix of the SARS-CoV-2 spike (S) 1; S1/S2-peptide mix of the spike (S) 1 and S2; S Sino-S1 protein (Sino Biological); M-peptide mix of the membrane; NC-peptide mix of the nucleocapsid; OI-T-SPOT.*COVID* (Oxford Immunotec).
